# Contribution of oxygen extraction fraction to maximal oxygen uptake in healthy young men

**DOI:** 10.1111/apha.13486

**Published:** 2020-05-30

**Authors:** Øyvind Skattebo, Jose A. L. Calbet, Bjarne Rud, Carlo Capelli, Jostein Hallén

**Affiliations:** ^1^ Department of Physical Performance Norwegian School of Sport Sciences Oslo Norway; ^2^ Department of Physical Education and Research Institute of Biomedical and Health Sciences (IUIBS) University of Las Palmas de Gran Canaria Gran Canaria Spain; ^3^ Department of Neurosciences, Biomedicine and Movement Sciences University of Verona Verona Italy

**Keywords:** arteriovenous oxygen difference, cardiac output, exercise, leg blood flow, limiting factors, maximal oxygen uptake, oxygen diffusion, stroke volume

## Abstract

We analysed the importance of systemic and peripheral arteriovenous O_2_ difference ( $a\text{-}\bar{v}O_{2}$ difference and a‐v_f_O_2_ difference, respectively) and O_2_ extraction fraction for maximal oxygen uptake ( ${\dot{V}O}_{\text{2max}}$). Fick law of diffusion and the Piiper and Scheid model were applied to investigate whether diffusion versus perfusion limitations vary with ${\dot{V}O}_{\text{2max}}$. Articles (*n* = 17) publishing individual data (*n* = 154) on ${\dot{V}O}_{\text{2max}}$, maximal cardiac output ( ${\dot{Q}}_{\max}$; indicator‐dilution or the Fick method), $a\text{-}\bar{v}O_{2}$ difference (catheters or the Fick equation) and systemic O_2_ extraction fraction were identified. For the peripheral responses, group‐mean data (articles: *n* = 27; subjects: *n* = 234) on leg blood flow (LBF; thermodilution), a‐v_f_O_2_ difference and O_2_ extraction fraction (arterial and femoral venous catheters) were obtained. ${\dot{Q}}_{\max}$ and two‐LBF increased linearly by 4.9‐6.0 L **·** min^–1^ per 1 L **·** min^–1^ increase in ${\dot{V}O}_{\text{2max}}$ (*R* ^2^ = .73 and *R* ^2^ = .67, respectively; both *P* < .001). The $a\text{-}\bar{v}O_{2}$ difference increased from 118‐168 mL **·** L^–1^ from a ${\dot{V}O}_{\text{2max}}$ of 2‐4.5 L **·** min^–1^ followed by a reduction (second‐order polynomial: *R* ^2^ = .27). After accounting for a hypoxemia‐induced decrease in arterial O_2_ content with increasing ${\dot{V}O}_{\text{2max}}$ (*R* ^2^ = .17; *P* < .001), systemic O_2_ extraction fraction increased up to ~90% ( ${\dot{V}O}_{\text{2max}}$: 4.5 L **·** min^–1^) with no further change (exponential decay model: *R* ^2^ = .42). Likewise, leg O_2_ extraction fraction increased with ${\dot{V}O}_{\text{2max}}$ to approach a maximal value of ~90‐95% (*R* ^2^ = .83). Muscle O_2_ diffusing capacity and the equilibration index *Y* increased linearly with ${\dot{V}O}_{\text{2max}}$ (*R* ^2^ = .77 and *R* ^2^ = .31, respectively; both *P* < .01), reflecting decreasing O_2_ diffusional limitations and accentuating O_2_ delivery limitations. In conclusion, although O_2_ delivery is the main limiting factor to ${\dot{V}O}_{\text{2max}}$, enhanced O_2_ extraction fraction (≥90%) contributes to the remarkably high ${\dot{V}O}_{\text{2max}}$ in endurance‐trained individuals.

**Abbreviations**
[Hb]: haemoglobin concentration
${\dot{Q}}_{\max}$: maximal cardiac output
${\bar{O}}_{2}$ extraction: systemic oxygen extraction fraction
${\dot{V}O}_{2}$: oxygen uptake
${\dot{V}O}_{\text{2max}}$: pulmonary maximal oxygen uptake
$a\text{-}\bar{v}O_{2}$ difference: arterial to mixed venous oxygen difference
$C\bar{v}O_{2}$: mixed venous oxygen content
a‐v_f_O_2_ difference: arterial to femoral venous oxygen difference
CaO_2_: arterial oxygen content
Cv_f_O_2_: femoral venous oxygen content
CVP: central venous pressure
D_M_O_2_: muscle O_2_ diffusing capacity
LBF: leg blood flow
Leg ${\dot{V}O}_{\text{2max}}$: maximal oxygen uptake of the leg
MAP: mean arterial blood pressure
MTT: erythrocyte capillary mean transit time
O_2_ extraction: peripheral (leg) oxygen extraction fraction
OXPHOS: maximal mitochondrial respiratory capacity
P_50_O_2_: partial pressure of O_2_ at 50% SO_2_
PO_2_: partial pressure of oxygen
SO_2_: oxygen saturation of haemoglobin

## 1 INTRODUCTION

Under resting conditions in humans, the O_2_ uptake ( ${\dot{V}O}_{2}$) is 3‐5 mL **·** kg^–1^ **·** min^–1^, and only a small fraction is consumed within the skeletal muscles.^1^ However, during incremental exercise, the pulmonary ${\dot{V}O}_{2}$ increases gradually and can reach a maximum ( ${\dot{V}O}_{\text{2max}}$) of ~90 mL **·** kg^–1^ **·** min^–1^ depending on gender, age, body weight, genetics, training status and health.^1^, ^2^, ^3^

According to the Fick equation, ${\dot{V}O}_{\text{2max}}$ is determined by the product of the maximal cardiac output ( ${\dot{Q}}_{\max}$) and the arterial to mixed venous O_2_ difference ( $a\text{-}\bar{v}O_{2}$ difference). ${\dot{Q}}_{\max}$ multiplied by the arterial O_2_ content (CaO_2_) sets the upper limit of systemic O_2_ delivery, which is the principal limitation to ${\dot{V}O}_{\text{2max}}$ during exercise recruiting a large muscle mass, at sea level.^4^, ^5^, ^6^ Despite extensive research since the 1950s on the factors limiting ${\dot{V}O}_{\text{2max}}$, it is still debated whether peripheral O_2_ extraction capacity contributes to limiting ${\dot{V}O}_{\text{2max}}$.^7^, ^8^ Several original studies^4^, ^5^, ^9^, ^10^, ^11^, ^12^ and review articles^6^, ^13^, ^14^, ^15^ have addressed this topic in recent decades, yet no study has aimed to statistically analyse all the existing data on the association between ${\dot{V}O}_{\text{2max}}$ and its limiting factors. This kind of analysis is warranted, as the original studies often used homogenous groups with a small number of subjects (<10) since they applied costly and invasive techniques involving catheterizations to determine ${\dot{Q}}_{\max}$ (indicator‐dilution techniques or the direct Fick method), regional blood flows (thermodilution or indicator‐dilution techniques) and O_2_ extraction fraction (calculated by the Fick equation or directly measured through arterial and venous catheters). Consequently, the statistical power is often too low to detect small but meaningful differences between subjects, groups with different training status and before and after training, thus precluding a definite conclusion.

It is documented that the $a\text{-}\bar{v}O_{2}$ difference at ${\dot{V}O}_{\text{2max}}$ is only slightly different between untrained and endurance‐trained individuals,^16^, ^17^ suggesting that peripheral adaptations to endurance training have only a minor impact on ${\dot{V}O}_{\text{2max}}$. However, the $a\text{-}\bar{v}O_{2}$ difference is determined not only by the peripheries’ ability to extract O_2_, reflected in the mixed venous O_2_ content ( $C\bar{v}O_{2}$) but also by the CaO_2_, which sets the upper limit for the $a\text{-}\bar{v}O_{2}$ difference during maximal exercise. The CaO_2_ is set by the haemoglobin concentration ([Hb]) and the O_2_ saturation of Hb (SO_2_), which may change with training and is acutely modified during exercise. For instance, endurance training causes plasma volume expansion^18^ that can lead to haemodilution and a lower O_2_ carrying capacity of the blood.^16^ A high ${\dot{Q}}_{\max}$ shortens the time for alveolar/capillary gas equilibration at the lung causing exercise‐induced arterial hypoxemia that further reduces the CaO_2_.^19^, ^20^ Therefore, it may be that the $a\text{-}\bar{v}O_{2}$ difference does not increase substantially after endurance training because of a concurrent training‐induced lowering of CaO_2_, whereas the systemic O_2_ extraction fraction ( ${\bar{O}}_{2}$ extraction: $a\text{-}\bar{v}O_{2}$ difference/CaO_2_) may improve.

Another aspect of this discussion is whether the measurement techniques are sensitive enough to detect meaningful changes in the $a\text{-}\bar{v}O_{2}$ difference. Most studies have not measured $a\text{-}\bar{v}O_{2}$ difference directly but calculated it using the Fick equation ( ${\dot{V}O}_{\text{2max}}$/ ${\dot{Q}}_{\max}$).^16^, ^17^, ^21^, ^22^, ^23^, ^24^ The reason why so few studies have measured $a\text{-}\bar{v}O_{2}$ difference directly during maximal exercise is because of the need for right heart catheterization. Therefore, studies measuring the arterial to femoral venous O_2_ difference (a‐v_f_O_2_ difference) and leg O_2_ extraction fraction directly using peripheral catheters may be more sensitive in evaluating whether the O_2_ extraction capacity changes with endurance training.

It is important to note that the factors limiting ${\dot{V}O}_{\text{2max}}$ may change over the course of training. For instance, the maximal mitochondrial respiratory capacity (OXPHOS) measured in permeabilized muscle fibres ex vivo and ${\dot{V}O}_{\text{2max}}$ is associated in untrained, but not in trained individuals.^25^ These and other data^26^ suggest that peripheral factors contribute to limit ${\dot{V}O}_{\text{2max}}$ in the untrained state, but their influence may diminish with increased ${\dot{V}O}_{\text{2max}}$ and training status.

In the present study, we critically reviewed and statistically analysed the previously published data on the association between ${\dot{V}O}_{\text{2max}}$ and O_2_ extraction fraction, in men, by focusing on catheterization studies. Two approaches were used: Part 1) articles containing individual data on pulmonary ${\dot{V}O}_{\text{2max}}$, ${\dot{Q}}_{\max}$ (indicator‐dilution techniques or the Fick method), $a\text{-}\bar{v}O_{2}$ difference (mostly calculated) and ${\bar{O}}_{2}$ extraction fraction measured during whole‐body maximal exercise (running, cycling) were included; Part 2) to investigate the relationship between limb ${\dot{V}O}_{2}$ and peripheral O_2_ extraction fraction, mean data from studies reporting leg blood flow (LBF), a‐v_f_O_2_ difference and leg O_2_ extraction fraction (catheters) measured during whole‐body maximal exercise (running, cycling, cross‐country skiing) were included. To investigate whether the limiting factors vary with ${\dot{V}O}_{\text{2max}}$, we employed the Fick law of diffusion to calculate the muscle O_2_ diffusing capacity (D_M_O_2_) and subsequently used the Piiper and Scheid model to calculate the relative roles of perfusion versus diffusion limitations to ${\dot{V}O}_{\text{2max}}$.^27^ Finally, we discuss the potential mechanisms behind the elevated O_2_ extraction fraction observed after endurance training.

## 2 ANALYSIS OF EXISTING DATA

The strategy to use individual and mean data to investigate the systemic and peripheral responses, respectively, was chosen since a large amount of individual data has been published on systemic responses, whereas we were unable to identify other than mean values in studies investigating peripheral haemodynamics and O_2_ extraction fraction. The data were identified through searches conducted in the PubMed database using several combinations of the following search terms: circulation, circulatory, hemodynamic(s), cardiac output, leg blood flow, arteriovenous oxygen difference, oxygen extraction and exercise. Cross‐reference checks were also conducted, in addition to separate searches on authors with articles already included in the database. Only exercise modes engaging a large muscle mass that could elicit ${\dot{V}O}_{\text{2max}}$ were included (cycling, running and cross‐country skiing using the diagonal technique). Data from cross‐sectional studies or before and after training interventions that were collected in normoxia on young (<40 years old) and healthy individuals were included. Data collected in hypoxia, after acclimatization to altitude, in altitude natives, in hyperthermia, with atrial pacing, after bed rest and after blood volume manipulations were excluded. The control condition was used when the above forms of manipulations of the cardiovascular system were conducted. Only catheterization studies that used invasive methods to measure ${\dot{Q}}_{\max}$ (indicator‐dilution techniques or the Fick method) and LBF (bolus or continuous infusion thermodilution and indicator‐dilution techniques) were included. Only individual data from men are used (Part 1). In Part 2, studies that had a sample with a majority of men were used (≥50%). When several papers reported data from the same data collection, only one of the articles was included. If an article used some of the same subjects as previously reported, but with supplementation with new subjects, the data were included. The included articles are presented in Tables 1 and 2 for Parts 1 and 2, respectively.

**Table 1:** Articles reporting individual values of maximal oxygen uptake ( ${\dot{V}O}_{\text{2max}}$), maximal cardiac output ( ${\dot{Q}}_{\max}$) and arterial to mixed venous O_2_ difference ( $a\text{-}\bar{v}O_{2}$ difference). In studies reporting arterial O_2_ content, the systemic O_2_ extraction fraction ( ${\bar{O}}_{2}$ extraction) was calculated

Article	*n*	Exercise	Age	Method used to measure:			Reported or can be calculated	
				${\dot{Q}}_{\max}$	${\dot{V}O}_{\text{2max}}$	$a\text{-}\bar{v}O_{2}$ difference	${\bar{O}}_{2}$ extraction	MAP
Blomqvist et al^116^	4	Cycling	23‐33	ID	DB	Calculated using Fick equation 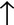	–	Yes
Ekblom and Hermansen^16^	14	Run	22‐34	ID	DB		Yes	Yes
Ekblom et al^17^	8	Cycling	19‐27	ID	DB		Yes	Yes
Ekblom^112^	7	Cycling	22‐26	ID	DB		Yes	Yes
Epstein et al^117^	2	Run	21	ID	Custom		–	Yes
Epstein et al^118^	4	Run	18‐30	Fick	Custom	Measured	–	Yes
Gleser^22^	6	Cycling	20‐23	ID	DB	Calculated using Fick equation 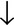	Yes	Yes
Hermansen et al^21^	13	Cycling/Run	19‐34	ID	DB		Yes	Yes
Mitchell et al^23^	6	Run	–	ID	DB		–	–
Robinson et al^119^	5	Run	19‐31	ID	Custom		–	Yes
Saltin^110^	4	Cycling	23‐26	ID	DB		–	–
Saltin and Stenberg^109^	4	Cycling	23‐25	ID	DB		–	Yes
Saltin et al^85^	5	Cycling	19‐21	ID	DB		Yes	Yes
Saltin et al^111^	4	Cycling	20‐21	ID	DB		–	–
Stenberg et al^114^	6	Cycling	20‐36	ID	DB		Yes	Yes
Stenberg et al^108^	5	Cycling	20‐39	ID	DB		–	Yes
Åstrand et al^24^	12	Cycling	21‐30	ID	DB		Yes	Yes

Abbreviations: DB, Douglas bag technique; ID, indicator‐dilution method using indocyanine green or Evans blue dye (only used in Mitchell et al^23^); MAP, mean arterial pressure; *n*, number of subjects meeting the inclusion criteria. Note that some subjects were investigated on more than one occasion (before/after training, running/cycling).

**Table 2:** Articles reporting mean values of leg oxygen uptake (leg ${\dot{V}O}_{\text{2max}}$), leg blood flow (LBF) and arterial to femoral venous O_2_ difference (a‐v_f_O_2_ difference) during maximal exercise (cycling and cross‐country skiing using the diagonal technique)

Article	*n*	Age ( $\bar{x}$)	Method used to measure:			Reported or can be calculated
			Pulmonary ${\dot{V}O}_{2}$	LBF	a‐v_f_O_2_ difference	O_2_ extraction
Bender et al^102^	7♂	22	Custom	TD‐B	Measured via arterial and femoral venous blood sampling 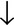	Yes
Calbet et al^57^	4♂3♀	24	Med. Graph. CPX	TD‐C		Yes
Calbet et al^29^, ^32^	3♂	24	Amis 2001	TD‐C		Yes
Calbet et al^52^	10♂	24	Quark b2	TD‐C		Yes
Calbet et al^5^	9♂	33	Quark b2	TD‐C		Yes
Calbet et al^31^	9♂	31	Quark b2	TD‐C		Yes
Calbet et al^56^	11♂	22	Vmax 29	TD‐C		Yes
Cardinale et al^10^	4♂3♀	33	Oxycon Pro	TD‐C		Yes
Cardus et al^104^	13♂5♀	23	Custom	TD‐C		Yes
Gonzalez‐Alonso et al^11^	8♂	24	OCM‐2	TD‐C		Yes
Harms et al^105^	7♂	29	Custom	TD‐C		Yes
Klausen et al^30^	6♂	23	Douglas bag tech.	ID‐B		–
Knight et al^103^	7♂	29	Custom	TD‐C		Yes
Knight et al^97^	12♂	29	Custom	TD‐C		Yes
Lundby et al^53^	8♂	26	Quark b2	TD‐C		Yes
Lundby et al^54^	8♂	27	Quark b2	TD‐C		Yes
Lundby et al^107^, ^113^	6♂	26	Custom	TD‐C		Yes
Mortensen et al^9^	13♂	28	Quark b2	TD‐C		Yes
Mortensen et al^4^	10♂	27	Quark b2	TD‐C		Yes
Munch et al^55^	10♂	27	Quark CPET	TD‐C		Yes
Poole et al^115^	6♂	26	Custom	TD‐C		Yes
Roca et al^28^	6♂	24	Custom	–		Yes
Roca et al^12^	8♂4♀	22	Custom	TD‐C		Yes
Proctor et al^120^	11♂	21	TrueMax 2400	TD‐C		Yes
Rud et al^45^	4♂4♀	23	Douglas bag tech.	TD‐C		Yes
Trangmar et al^76^	9♂	26	Not reported	TD‐C		Yes
van Hall et al^106^	5♂1♀	26	Med. Graph. CPX	TD‐C		Yes

Abbreviations: ID‐B, bolus indicator‐dilution method (I‐labelled human albumin); *n*, number of subjects; TD‐B, bolus‐infusion thermodilution method; TD‐C, continuous‐infusion TD.

### 2.1 Calculations

When the data were published in graphs and not in tables or text, ImageJ (v1.50b; National Institutes of Health, USA) was used for data extraction. If not all variables were reported in the articles, the reported data were used to derive the missing values via the following formulas or combination of formulas if possible:

(1) $$\text{Pulmonary}\;{\dot{V}O}_{\text{2max}}={\dot{Q}}_{\max}\times a\text{-}\bar{v}O_{2}\;\text{difference}$$

(2) $$\text{Leg}\;{\dot{V}O}_{\text{2max}}=\;\text{LBF}\times {\;\text{a-v}}_{f}O_{2}\;\text{difference}$$

(3) $$\text{Stroke volume}\;={\dot{Q}}_{\max}/\text{heart rate}$$

(4) $${\text{Blood O}}_{2}\;\text{content}\;\left(e\text{.g}{\text{., CaO}}_{2}\right)\;=1.39\times \;\left[\text{Hb}\right]\;\times {\;\text{SO}}_{2}+\;0.003\times {\;\text{PO}}_{2}$$

(5) $${\text{Leg O}}_{2}\;\text{delivery}\;=\;\text{LBF}\;\times {\;\text{CaO}}_{2}$$

(6) $${\bar{O}}_{2}\;\text{extraction}\;=a\text{-}\bar{v}O_{2}\;\text{difference}/{\text{CaO}}_{2}$$

(7) $$O_{2}\;\text{extraction}\;={\;\text{a-v}}_{f}O_{2}\;\text{difference}/{\text{CaO}}_{2}$$

(8) $$\text{Systemic vascular conductance}\;={\dot{Q}}_{\max}/\left(\text{MAP}-\text{CVP}\right)$$

If no arterial partial pressure of O_2_ (PO_2_) was reported, 100 mmHg was assumed for the calculation of CaO_2_ (ie, 3 mL O_2_ freely dissolved in blood plasma per 1 L of blood). Central venous pressure (CVP) at ${\dot{V}O}_{\text{2max}}$ was taken as 5 mmHg^4^ when calculating systemic vascular conductance. D_M_O_2_ and mean capillary PO_2_ were calculated as previously described,^28^, ^29^ using the measured arterial and femoral venous PO_2_. D_M_O_2_ [D_M_O_2_ = ${\dot{V}O}_{2}$/(mean capillary PO_2_—mitochondrial PO_2_); ie, the O_2_ conductance from the capillary to the mitochondria] is recognized as a compound variable integrating several steps in the O_2_ cascade, including the dissociation of O_2_ from Hb, and diffusion through the erythrocyte membrane, plasma, capillary wall, interstitial space, sarcolemma, cytoplasm (myoglobin facilitated or by diffusion) and into the mitochondria for utilization by the cytochromes. The equilibration index *Y*, which quantitatively describes perfusion versus diffusion limitations to ${\dot{V}O}_{2}$, was calculated according to Piiper and Scheid.^27^

### 2.2 Statistical analyses

Data are presented as mean±standard deviation, if not otherwise stated. Regression was analysed using simple linear regression, second‐order polynomials and exponential decay models (*y* = *a* **·** *e* ^‐^ *^K^* ^**·**^ *^X^*+plateau), all using least squares as the fitting method. Regression lines/curves are presented with 95% confidence bands representing the likely location of the true curve. The alpha‐level was set to≤.05 and values between>.05 and≤.10 were considered to indicate trends. GraphPad Prism (v. 8.0.1; GraphPad Software, CA, USA) and Microsoft Office Excel 2013 (Microsoft Corporation, WA, USA) were used for statistical analysis.

### 2.3 Part 1: Systemic responses during maximal exercise (individual data)

**Figure 1:**
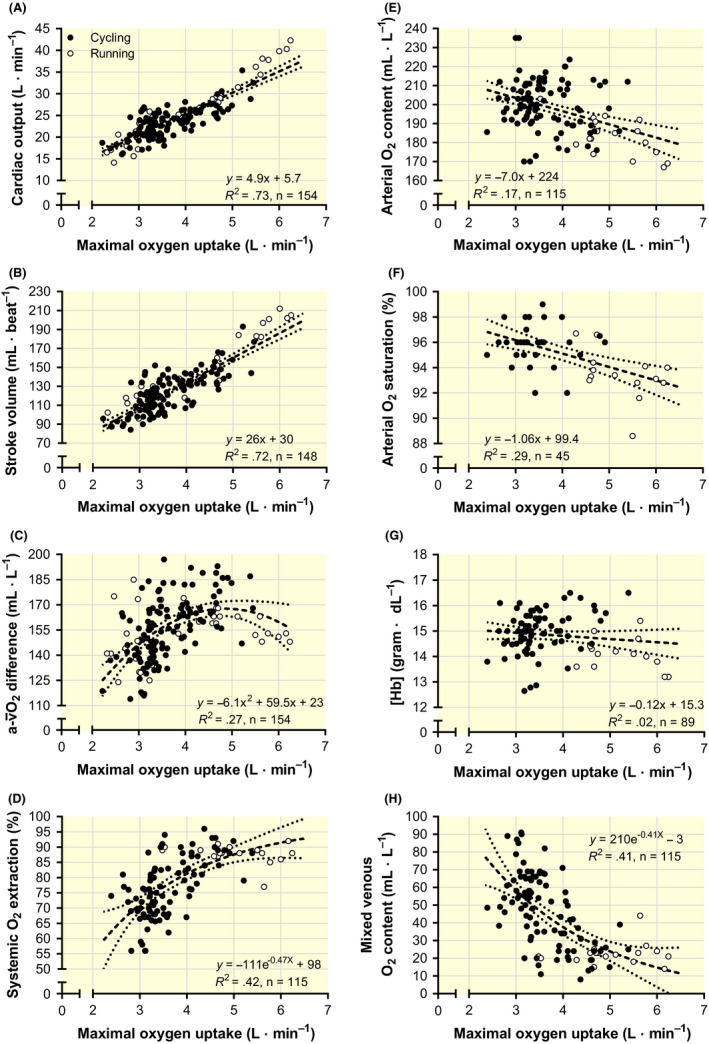
The relationship between individual values (from studies reported in Table 1) of pulmonary maximal oxygen uptake and cardiac output (A), stroke volume (B), arterial to mixed venous oxygen difference ( $a\text{-}\bar{v}O_{2}$ difference; C), systemic oxygen extraction fraction (D), arterial oxygen content (E), arterial oxygen saturation (F), haemoglobin concentration ([Hb]; G) and the calculated mixed venous oxygen content (H). All data were obtained during maximal exercise. Inserted in each graph are the formulas for the regression equations along with the goodness of fit (*R* ^2^) and the number of data pairs (*n*)

${\dot{Q}}_{\max}$ increased by 4.9 L**·**min^−1^ for each L**·**min^−1^ increase in ${\dot{V}O}_{\text{2max}}$ (Figure 1A; *P* < .001), explained by a linear increase in stroke volume (Figure 1B; *P* < .001).

The calculated $a\text{-}\bar{v}O_{2}$ difference ( ${\dot{V}O}_{\text{2max}}$/ ${\dot{Q}}_{\max}$) showed an inverse J‐shaped curve, reaching the highest level between 4.5‐5.0 L**·**min^−1^ before declining at higher ${\dot{V}O}_{\text{2max}}$ (Figure 1C). After accounting for the decrease in CaO_2_ with increasing ${\dot{V}O}_{\text{2max}}$ (Figure 1E; *P* < .001), the calculated ${\bar{O}}_{2}$ extraction fraction increased up to a ${\dot{V}O}_{\text{2max}}$ of ~4.5‐5.0 L**·**min^−1^ and then approached a maximal value at ~90% (Figure 1D) when restricting the exponential decay model to plausible physiological limits ( ${\dot{V}O}_{\text{2max}}$: 6‐7 L**·**min^−1^). The linear decrease in CaO_2_ was explained by arterial hypoxemia (decreased arterial SO_2_; Figure 1F; *P* < .001) and a non‐significant negative relationship between [Hb] and ${\dot{V}O}_{\text{2max}}$ (Figure 1G; *P* = .232). The calculated $C\bar{v}O_{2}$ gradually decayed and approached a minimum at ~10‐15 mL **·** L^–1^ in the subjects with the highest ${\dot{V}O}_{\text{2max}}$ (Figure 1H).

**Figure 2:**
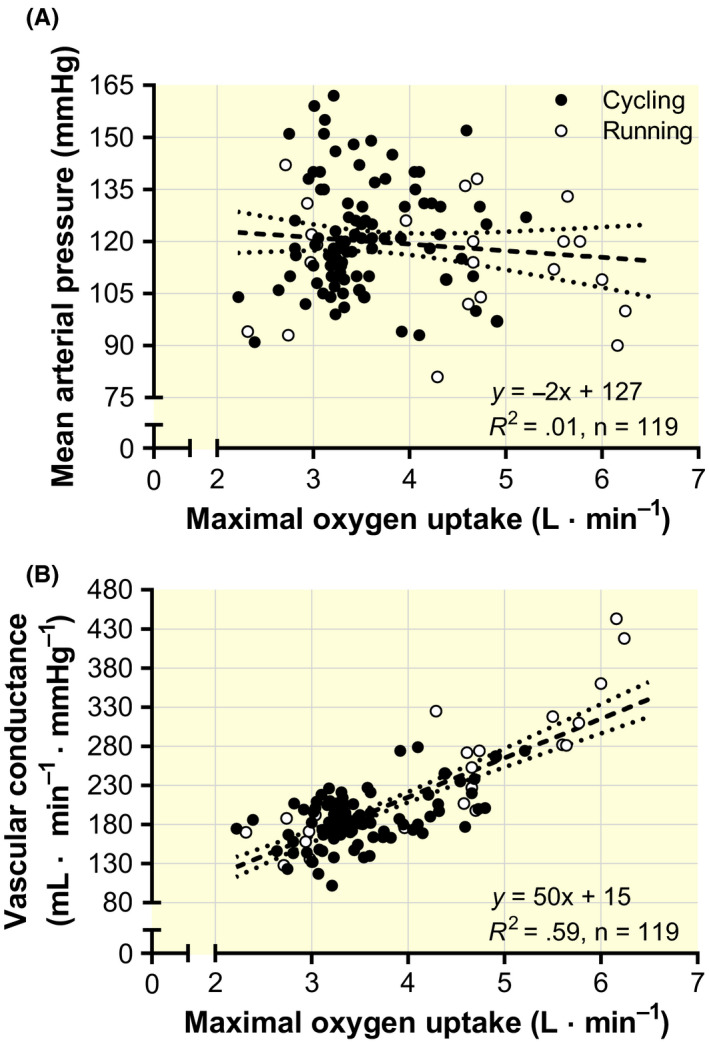
The relationship between individual values (from studies reported in Table 1) of pulmonary maximal oxygen uptake and mean arterial pressure (A) and systemic vascular conductance (B). Inserted in each graph are the formulas for the linear regression along with the goodness of fit (*R* ^2^) and the number of data pairs (*n*)

Systemic vascular conductance was strongly positively correlated with ${\dot{V}O}_{\text{2max}}$ (Figure 2B; *P* < .001). There were no significant associations between mean arterial pressure (MAP) and ${\dot{V}O}_{\text{2max}}$ (Figure 2A; *P* = .289) or with ${\dot{Q}}_{\max}$ (*y* = −0.2*x* + 125; *R* ^2^ = .004; *n* = 119; *P* = .475).

**Figure 3:**
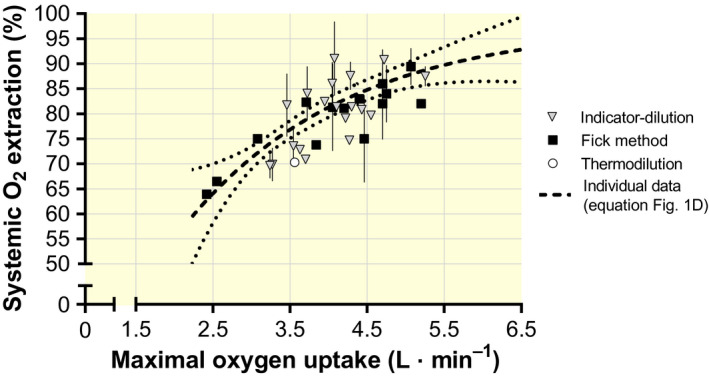
Mean values (±95% confidence limits, where available) of systemic oxygen extraction fraction versus maximal oxygen uptake from studies using the direct (pulmonary artery catheter) or the modified (right atrium catheter) Fick method,^4^, ^9^, ^28^, ^32^, ^51^, ^55^, ^61^, ^121^, ^122^, ^123^, ^124^, ^125^, ^126^, ^127^ the indicator dilution method^5^, ^11^, ^16^, ^17^, ^21^, ^22^, ^24^, ^31^, ^42^, ^52^, ^53^, ^54^, ^57^, ^85^, ^112^, ^114^, ^128^, ^129^, ^130^ and the transpulmonary thermodilution method.^56^ Broken line is the regression equation obtained from Figure 1D

When controlling the regression between the individual data of ${\dot{V}O}_{\text{2max}}$ and the calculated ${\bar{O}}_{2}$ extraction fraction with mean values from studies measuring ${\bar{O}}_{2}$ extraction fraction directly using the Fick method (right heart catheterization), or indirectly using the Fick equation ( ${\dot{Q}}_{\max}$: indicator‐dilution or transpulmonary thermodilution), most values fell close to the regression curve (Figure 3).

### 2.4 Part 2: Peripheral responses during maximal exercise (mean data)

**Figure 4:**
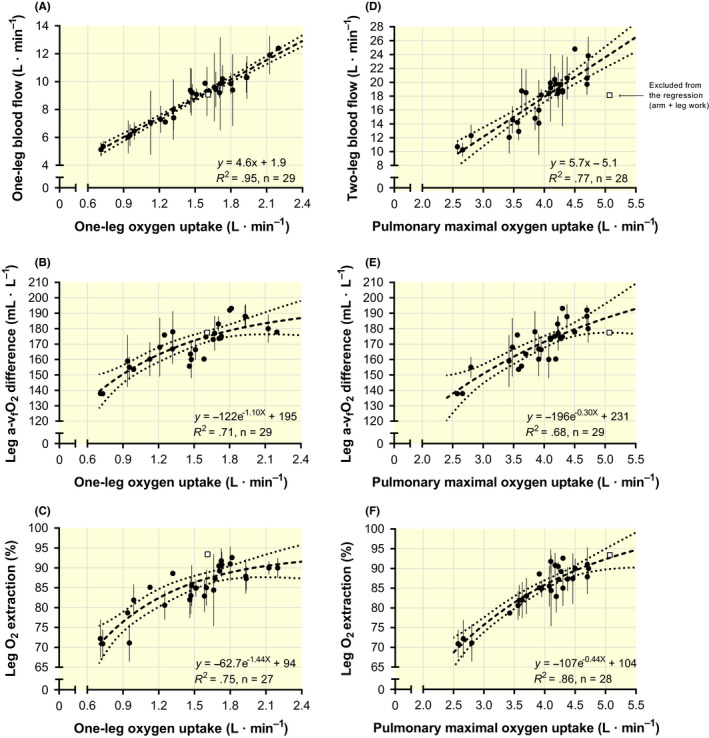
The relationship between one‐leg or pulmonary maximal oxygen uptake and leg blood flow (Figure 4A,D, respectively), arterial to femoral venous oxygen difference (a‐v_f_O_2_ difference; Figure 4B,E, respectively) and leg oxygen extraction fraction (Figure 4C,F, respectively). Black circles and white squares denote cycling and diagonal cross‐country skiing respectively. The skiers are excluded from the regression in Figure 4D owing to the combined leg and arm use for locomotion that distributed 6.6 L**·**min^−1^ blood flow to the exercising arms (see the discussion). Data are mean values (±95% confidence limits, where available) from studies reported in Table 2

LBF and two‐LBF rose by 4.6 and 5.7 L**·**min^−1^ for each L**·**min^−1^ increase in leg and pulmonary ${\dot{V}O}_{\text{2max}}$ respectively (Figure 4A,D; both *P* < .001). Leg and pulmonary ${\dot{V}O}_{\text{2max}}$ displayed a linear relationship (*y* = 1.27*x* – 2.01; *R* ^2^ = .85; *n* = 28; *P* < .001). The directly measured leg a‐v_f_O_2_ difference and leg O_2_ extraction fraction were best explained by exponential decay models and increased gradually with the increase in leg and pulmonary ${\dot{V}O}_{\text{2max}}$ to approach a maximum at ~180‐190 mL **·** L^–1^ and ~90‐95% respectively (Figure 4B,C,E,F). These relationships were equally strong when ${\dot{V}O}_{\text{2max}}$ was standardized to body weight (Supporting material Figure 1). Note that leg a‐v_f_O_2_ difference was not lower for the subjects with the highest ${\dot{V}O}_{\text{2max}}$, as observed for the systemic $a\text{-}\bar{v}O_{2}$ difference (Figure 1C), possibly since only one subject group exceeded a ${\dot{V}O}_{\text{2max}}$ of 4.7 L**·**min^−1^, where this occurred for the systemic responses (see Figure 1C). In connection, no association was evident between pulmonary ${\dot{V}O}_{\text{2max}}$ and CaO_2_ for these data (*y* = 1.07 + 195; *R* ^2^ < .01; *n* = 30; *P* = .701).

**Figure 5:**
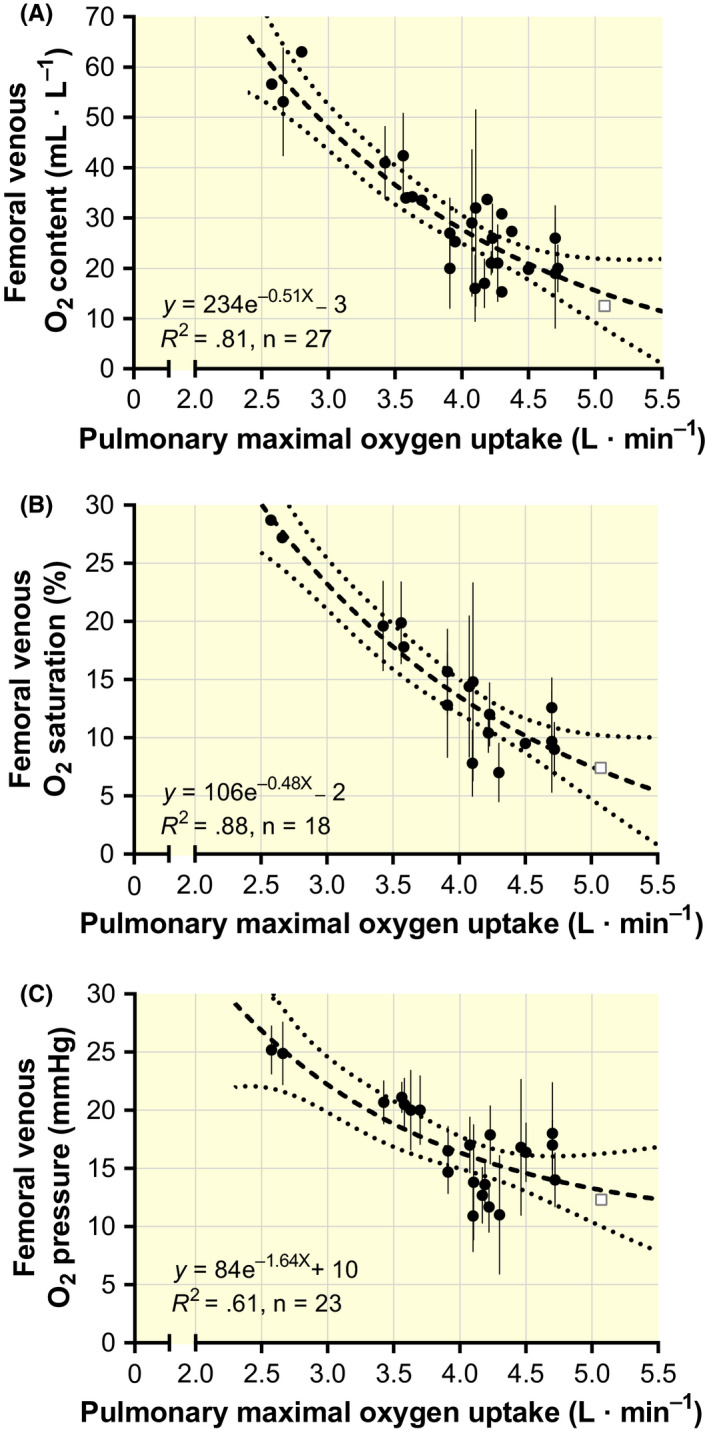
The relationship between pulmonary maximal oxygen uptake and the associated femoral venous O_2_ content (A), femoral venous O_2_ saturation (B) and femoral venous O_2_ pressure (C). Black and white symbols denote cycling and diagonal skiing, respectively. Data are mean values (±95% confidence limits, where available) from studies reported in Table 2

Like the systemic responses, the measured femoral venous O_2_ content (Cv_f_O_2_) decreased gradually with increasing pulmonary ${\dot{V}O}_{\text{2max}}$ until reaching a minimum of ~10 mL **·** L^–1^ (Figure 5A). Likewise, the femoral venous SO_2_ and PO_2_ decreased gradually to approach ~5% and ~10 mmHg at the highest ${\dot{V}O}_{\text{2max}}$ respectively (Figure 5B,C).

**Figure 6:**
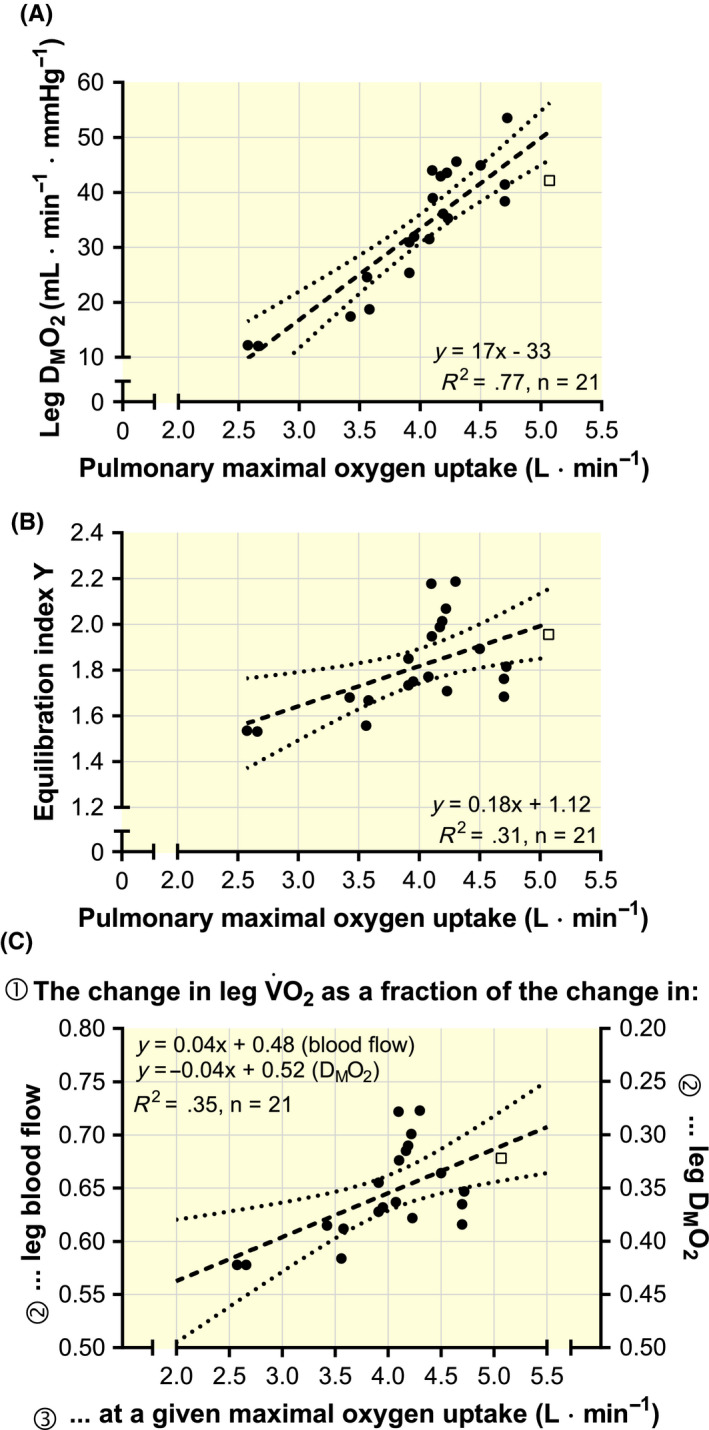
The relationship between pulmonary maximal oxygen uptake ( ${\dot{V}O}_{\text{2max}}$) and one‐leg muscle O_2_ diffusing capacity (D_M_O_2_; A), the equilibration index *Y* (B), calculated using the Piiper and Scheid model, and the fractional extent to which ${\dot{V}O}_{\text{2max}}$ is expected to change if D_M_O_2_ or leg blood flow (LBF) is changed alone (C).^27^ Data are mean values from studies reported in Table 2

D_M_O_2_ was positively correlated with leg ${\dot{V}O}_{\text{2max}}$ (*y* = 27*x* – 6; *R* ^2^ = .92; *n* = 21; *P* < .001), pulmonary ${\dot{V}O}_{\text{2max}}$ (Figure 6A; *P* < .001) and leg O_2_ extraction fraction (*y* = 1.7*x* – 110; *R* ^2^ = .80; *n* = 21; *P* < .001). Interestingly, the equilibration index *Y*, which quantitatively describes diffusion versus perfusion limitations to muscle ${\dot{V}O}_{2}$ (where *Y* < 0.1 indicates pure diffusion limitation, 0.1 < *Y* < 3 indicates mixed perfusion‐diffusion limitation and *Y* > 3 indicates pure perfusion limitation),^27^ was well above 1.0 for all subject groups (Figure 6B) and increased progressively with leg ${\dot{V}O}_{\text{2max}}$ (*y* = 0.28*x* + 1.40; *R* ^2^ = .37; *n* = 21; *P* = .003), pulmonary ${\dot{V}O}_{\text{2max}}$ (Figure 6B; *P* = .008) and leg O_2_ extraction fraction (*y* = 0.023*x* – 0.129; *R* ^2^ = .53; *n* = 21; *P* < .001). The equilibration index *Y* was also correlated with pulmonary ${\dot{V}O}_{\text{2max}}$ standardized to body weight (*R* ^2^ = .38; *P* = .003; Supporting material Figure 2). Therefore, the leg muscles were more perfusion than diffusion limited, even for subjects with the lowest ${\dot{V}O}_{\text{2max}}$, and were progressively more perfusion/O_2_ delivery limited with a gradually higher ${\dot{V}O}_{\text{2max}}$. This can also be illustrated by applying the Piiper and Scheid model to calculate the fractional extent to which ${\dot{V}O}_{\text{2max}}$ is expected to change if D_M_O_2_ or LBF are modified^27^; Figure 6C shows that an individual’s ${\dot{V}O}_{\text{2max}}$ is less sensitive to any change in D_M_O_2_ if the ${\dot{V}O}_{\text{2max}}$ is already high, which is caused by the little remaining O_2_ available for extraction in the femoral venous (ie, end‐capillary) blood. For instance, according to this theoretical model and using the relationship in Figure 6C; if a subject with a ${\dot{V}O}_{\text{2max}}$ of 5 L**·**min^−1^ changed his D_M_O_2_ by 20%, he would only change his ${\dot{V}O}_{\text{2max}}$ by ~6% (20% × 0.3). Conversely, the same subject would increase ${\dot{V}O}_{\text{2max}}$ by ~14% after a 20% increase in LBF (20% × 0.7).

## 3 SUMMARY OF FINDINGS

To our knowledge, the present investigation is the first to critically review the existing research on the association between ${\dot{V}O}_{\text{2max}}$ and systemic and peripheral O_2_ extraction fractions in healthy young men. Our findings are as follows:

1. Pulmonary and leg ${\dot{V}O}_{\text{2max}}$ were best explained by ${\dot{Q}}_{\max}$ and LBF, respectively, agreeing with most previous studies where these variables have been directly manipulated.
2. The systemic ${\bar{O}}_{2}$ extraction fraction increased with ${\dot{V}O}_{\text{2max}}$ until approximately 4.5‐5.0 L**·**min^−1^. Above this value, the ${\bar{O}}_{2}$ extraction fraction was typically around ~90%.
3. The measured leg O_2_ extraction fraction increased with leg and pulmonary ${\dot{V}O}_{\text{2max}}$ to approach a maximal value at ~90‐95%, strengthening the findings from the calculated systemic ${\bar{O}}_{2}$ extraction fraction. This strongly suggests that O_2_ extraction increases after endurance training and contributes to a high ${\dot{V}O}_{\text{2max}}$.
4. The calculated $C\bar{v}O_{2}$ and the measured Cv_f_O_2_ indicate a minimum value at ~15 and ~10 mL **·** L^–1^, respectively, associated with a femoral venous SO_2_ and PO_2_ of ~5% and ~10 mmHg respectively. At this point, further peripheral O_2_ extraction may no longer be possible as a result of diffusional limitations and/or because the remaining O_2_ represents blood perfusing the least active muscle regions of the leg, connective tissue, bone marrow, adipose tissue and skin, which are characterized by a lower O_2_ extraction.
5. The progressive increase in the equilibration index *Y* with pulmonary and leg ${\dot{V}O}_{\text{2max}}$ indicates that the muscles become gradually more perfusion/O_2_ delivery limited with increasing ${\dot{V}O}_{\text{2max}}$.

### 3.1 Oxygen delivery

To match O_2_ delivery to O_2_ consumption, ${\dot{Q}}_{\max}$ and two‐LBF increased by ~5‐6 L**·**min^−1^ per 1 L**·**min^−1^ increase in pulmonary ${\dot{V}O}_{\text{2max}}$. These relationships were strong and complied with previous research and the “classic” view that O_2_ delivery is the primary determinant of whole‐body ${\dot{V}O}_{\text{2max}}$.^4^, ^7^, ^11^ As maximal heart rate showed no apparent relationship with ${\dot{V}O}_{\text{2max}}$, the high stroke volumes (>180 mL **·** beat^–1^) explained the large ${\dot{Q}}_{\max}$ in the athletes included in the present analysis (>35 L**·**min^−1^), in agreement with previous knowledge.^13^, ^16^, ^30^

Despite increased ${\dot{Q}}_{\max}$, MAP was unchanged with increasing ${\dot{V}O}_{\text{2max}}$ as a result of increased vascular conductance. Although untrained individuals typically display a rise in MAP from rest to maximal exercise,^31^ well‐trained athletes can display an unchanged MAP or even a small reduction owing to profound peripheral vasodilation.^32^ Consequently, vasodilation of a well‐developed peripheral vascular network likely contributed to the extremely high stroke volumes by minimizing afterload in the subjects with the highest ${\dot{V}O}_{\text{2max}}$. To substantiate, endurance training of each leg separately, to evoke extensive peripheral adaptations without stimulating the central circulation substantially, has been shown to decrease MAP and the total peripheral resistance during two‐legged maximal exercise that likely contributed to the elevated stroke volume and ${\dot{Q}}_{\max}$ after training.^30^ The high stroke volumes are probably achieved through the combined effect of a large left ventricular mass,^33^, ^34^ compliant cardiac chambers^35^, ^36^ and an expanded blood volume^37^, ^38^ that facilitates a high end‐diastolic volume and preload combined with the relatively low afterload.

### 3.2 Oxygen extraction

The calculated systemic $a\text{-}\bar{v}O_{2}$ difference showed a large variability for a given ${\dot{V}O}_{\text{2max}}$ and was, if anything, lower in those subjects displaying the highest ${\dot{V}O}_{\text{2max}}$ (>5 L**·**min^−1^) compared to those being moderately to well trained ( ${\dot{V}O}_{\text{2max}}$: 4‐5 L**·**min^−1^). This agrees with previous studies showing only a small difference between non‐endurance‐trained and active individuals^16^, ^17^ and no apparent difference between well‐trained individuals and elite athletes.^16^ This has led previous investigators to argue that improved O_2_ extraction does not contribute or only minimally contributes to the remarkably high ${\dot{V}O}_{\text{2max}}$ observed in elite athletes.^14^, ^39^ However, these papers may not have considered that endurance training causes plasma volume expansion,^18^ which often leads to haemodilution and a lower O_2_ carrying capacity of the arterial blood.^16^ Combined with the below‐average haemoconcentration from rest to maximal exercise that occurs in well‐trained individuals^16^ and the exercise‐induced arterial hypoxemia that often accompanies a high ${\dot{Q}}_{\max}$,^19^, ^20^ individuals with the highest ${\dot{V}O}_{\text{2max}}$ displayed a substantially lower CaO_2_ (~10%) than those with a low ${\dot{V}O}_{\text{2max}}$ (<180 mL **·** L^–1^ vs >200 mL **·** L^–1^; Figure 1E). Therefore, the lower CaO_2_ may explain why moderately and well‐trained individuals can have a similar $a\text{-}\bar{v}O_{2}$ difference, despite differing markedly in D_M_O_2_, mitochondrial mass and capillary density.^40^, ^41^ Actually, parts of this mechanism are demonstrated experimentally since acute plasma volume expansion increases ${\dot{Q}}_{\max}$ but lowers the CaO_2_ and, hence, reduces the $a\text{-}\bar{v}O_{2}$ difference during maximal exercise.^42^, ^43^

Opposite to the $a\text{-}\bar{v}O_{2}$ difference, the systemic ${\bar{O}}_{2}$ extraction fraction—ie, the fraction of O_2_ that is taken up with respect to the amount available for utilization ( $a\text{-}\bar{v}O_{2}$ difference/CaO_2_)—increased with ${\dot{V}O}_{\text{2max}}$ until reaching ~90%. This pattern was confirmed in the leg when measured using catheters, with the O_2_ extraction fraction increasing progressively with leg and pulmonary ${\dot{V}O}_{\text{2max}}$ until reaching ~90 to 95%. Therefore, the calculated systemic ${\bar{O}}_{2}$ extraction fraction (Fick equation) is supported by direct measurements via arterial and femoral venous blood sampling and strongly indicates that the ${\bar{O}}_{2}$ extraction fraction is improved with increasing ${\dot{V}O}_{\text{2max}}$ to a certain level.

In most endurance training studies investigating the interplay between central and peripheral adaptations in improving ${\dot{V}O}_{\text{2max}}$, ${\dot{Q}}_{\max}$ was measured by non‐invasive methods (such as inert‐gas rebreathing techniques, impedance cardiography and bioreactance) and the Fick equation was used to derive the $a\text{-}\bar{v}O_{2}$ difference (for references, see the meta‐analysis by Montero et al^44^). The majority of these studies failed to detect a statistically significant change in the $a\text{-}\bar{v}O_{2}$ difference. However, this finding does not necessarily mean that ${\dot{V}O}_{\text{2max}}$ was exclusively increased by elevated ${\dot{Q}}_{\max}$ for three reasons. First, when the $a\text{-}\bar{v}O_{2}$ difference is calculated by the Fick equation, a large variation is introduced as a result of measurement error in ${\dot{Q}}_{\max}$, especially when non‐invasive methods are used. Second, because of the above, maybe in combination with a considerable individual variation in peripheral adaptations such as capillarization, it is likely that these studies are underpowered for detecting small changes in the $a\text{-}\bar{v}O_{2}$ difference. Third, these studies may have failed to detect actual improvements in systemic ${\bar{O}}_{2}$ extraction fraction when the $a\text{-}\bar{v}O_{2}$ difference was mostly unchanged, as endurance training may have evoked an accompanying reduction in CaO_2_. Therefore, future studies should strive to measure peripheral or systemic O_2_ extraction fraction directly, or at least combine the calculations of $a\text{-}\bar{v}O_{2}$ difference with measurement of CaO_2_ (arterial catheter). Actually, in the endurance training studies where peripheral O_2_ extraction fraction was measured directly during maximal exercise (arterial and venous catheters), the vast majority found an increased O_2_ extraction fraction after training.^12^, ^30^, ^45^, ^46^, ^47^

A particular case, concerning the relationship between one‐leg ${\dot{V}O}_{\text{2max}}$ and O_2_ extraction fraction (Figure 4C) and between pulmonary ${\dot{V}O}_{\text{2max}}$ and two‐LBF (Figure 4D) deserves some attention (the white squares). These data were collected during combined upper‐ and lower‐body exercise (cross‐country skiing using the diagonal technique) and 6.6 L**·**min^−1^ of ${\dot{Q}}_{\max}$ was distributed to the two arms.^32^ Hence, when combining the locomotor blood flow (arms+legs), the data fall perfectly on the regression line between blood flow and pulmonary ${\dot{V}O}_{\text{2max}}$. When redistributing LBF towards other exercising musculature, the erythrocyte capillary mean transit time (MTT) is increased. Therefore, the conditions for Hb‐O_2_ off‐loading are improved, resulting in a slightly higher O_2_ extraction fraction for a given leg ${\dot{V}O}_{2}$. The same phenomenon can be seen when adding arm cycling to ongoing leg cycling^48^ or vice versa,^49^ which increases the O_2_ extraction fraction that compensates for some of the reduction in blood flow.

### 3.3 Limitations to ${\dot{V}O}_{\text{2max}}$ by O_2_ delivery and O_2_ extraction varies with training status

The equilibration index *Y* was positively correlated with ${\dot{V}O}_{\text{2max}}$. Therefore, endurance training leads to a situation where the muscles become gradually more O_2_‐delivery limited. Thus, individuals with the highest ${\dot{V}O}_{\text{2max}}$ can only achieve a further substantial improvement in ${\dot{V}O}_{\text{2max}}$ by increasing O_2_ delivery, a conclusion supported by the extremely low levels of Cv_f_O_2_ and $C\bar{v}O_{2}$ in these subjects. Therefore, the limiting factors to ${\dot{V}O}_{\text{2max}}$ change with training status and ${\dot{V}O}_{\text{2max}}$: (a) untrained, but healthy individuals display mixed perfusion‐diffusion limitations; and (b) this diffusional limitation reduces as ${\dot{V}O}_{\text{2max}}$ is increased.^26^ These conclusions are similar to those of Gifford et al,^25^ who found a clear relationship between OXPHOS measured in permeabilized muscle fibres ex vivo and ${\dot{V}O}_{\text{2max}}$ in untrained but not in trained individuals.

### 3.4 Why is not all the O_2_ extracted from the blood?

The entire ${\dot{Q}}_{\max}$ cannot be directed to the skeletal muscles during exercise. Other organs like the brain, heart, splanchnic organs and skin need perfusion and O_2_ delivery to maintain homeostasis. ${\dot{Q}}_{\max}$ must also serve the O_2_ demand of the respiratory muscles and the muscles in the trunk and the arms that stabilize the subject’s position on the cycle ergometer, and these tissues are characterized by a substantially lower O_2_ extraction than the legs during maximal exercise.^5^, ^50^ As a mean of those investigations measuring ${\dot{Q}}_{\max}$ and LBF simultaneously (Table 3), the non‐leg blood flow was 6.4 L**·**min^−1^ and was unaffected by the level of ${\dot{Q}}_{\max}$ (*y* = 0.002*x* + 6.4; *R* ^2^ < .001; *n* = 12; *P* > .999).^4^, ^5^, ^9^, ^11^, ^31^, ^51^, ^52^, ^53^, ^54^, ^55^, ^56^, ^57^ The O_2_ extraction was calculated to be 68% on average for all non‐leg tissues (head, trunk and arms), explaining why the ${\bar{O}}_{2}$ extraction fraction of the central circulation was slightly lower than in the legs (79% vs 84%, respectively; Table 3). A mean difference of 5 percentage points might be a small underestimation since the studies using right heart catheterization^4^, ^9^, ^28^, ^29^, ^51^, ^55^ combined with arterial and femoral venous catheters indicated a mean difference of 8 percentage points. A difference of 5%‐8% points fits well, since the O_2_ extraction fraction of the arms, myocardium, brain and trunk range from 40% to 80% during exercise.^5^, ^50^, ^58^, ^59^, ^60^ Therefore, the $C\bar{v}O_{2}$ can never reach the same level as the Cv_f_O_2_ during exercise involving the legs and was calculated to reach a minimum of ~15 mL **·** L^–1^ in subjects having a ${\dot{V}O}_{\text{2max}}$ of 6 L**·**min^−1^ (Figure 1H). To our knowledge, the lowest $C\bar{v}O_{2}$ measured at sea level using right heart (atrium) catheterization is 20.1 mL **·** L^–1^ (group mean) in athletes with a ${\dot{V}O}_{\text{2max}}$ of 5.1 L**·**min^−1^.^29^ A slightly lower value was measured in one of these cross‐country skiers (15.5 mL **·** L^–1^), and a mean value of 18.6 mL **·** L^–1^ has been measured in moderately trained individuals after acclimatizing to 6500 metres above sea level^61^; indicating that 15 mL **·** L^–1^ or lower is approachable.

**Table 3:** Data from studies measuring pulmonary O_2_ uptake, cardiac output (indicator‐dilution, Fick method or transpulmonary thermodilution), leg blood flow (thermodilution) and leg arteriovenous O_2_ difference (a‐vO_2_ difference; catheters) simultaneously during maximal exercise. From these measurements, O_2_ extraction fraction was calculated for the central circulation and the non‐leg tissue (combined trunk, arms and head)

	Central circulation (mean ± SD)	Two‐leg circulation (mean ± SD)	Non‐leg tissue circulation (mean ± SD)
Blood flow (L**·**min^−1^)	25.0 ± 2.4	18.6 ± 3.0	6.4 ± 1.7
Arterial O_2_ content (mL **·** L^–1^)	203 ± 10	203 ± 10	203 ± 10
O_2_ delivery (L**·**min^−1^)	5.03 ± 0.60	3.77 ± 0.63	1.26 ± 0.32
O_2_ uptake (L**·**min^−1^)	4.02 ± 0.65	3.19 ± 0.65	0.83 ± 0.24
a‐vO_2_ difference (mL **·** L^–1^)	160 ± 17	172 ± 14	137 ± 48
O_2_ extraction fraction (%)	79 ± 8	84 ± 5	68 ± 26
Venous O_2_ content (mL **·** L^–1^)	42 ± 18	31 ± 10	66 ± 52
O_2_ delivery not utilized (L**·**min^−1^)	1.01 ± 0.36	0.58 ± 0.07	0.43 ± 0.32

Note

*n* = 12 (articles)^4^, ^5^, ^9^, ^11^, ^31^, ^51^, ^52^, ^53^, ^54^, ^55^, ^56^, ^57^ or *n* = 117 (subjects).

The highest recorded leg O_2_ extraction fraction was 93% (group mean)^29^ and the regression models indicated a plateau at ~95% within physiological limits for pulmonary ${\dot{V}O}_{\text{2max}}$. Hence, a minimum of ~10 mL O_2_ remains in each litre of femoral venous blood associated with a PO_2_ of ~10 mmHg, even for the best trained individuals. In this situation, a PO_2_ gradient persists between the blood and myoglobin (myoglobin/intracellular PO_2_: ~1‐2 mmHg),^62^ where myoglobin‐facilitated diffusion should proceed given the high myoglobin O_2_ affinity (myoglobin P_50_O_2_: ~5 mmHg) and the low myoglobin SO_2_ at maximal exercise.^62^ However, according to the Fick law of diffusion, the diffusive flux is directly proportional to the PO_2_ gradient and will, thus, gradually decrease along the capillary and be very small when approaching low capillary PO_2_ values such as 10 mmHg. It has also been shown that the primary site of resistance to O_2_ diffusion is between the capillaries and the sarcoplasm and it has been estimated that the “critical capillary PO_2_” needed to overcome this resistance may be as high as 10‐20 mmHg.^62^, ^63^, ^64^, ^65^ The remaining O_2_ may, therefore, represent diffusional limitations across the combined capillary wall, interstitium and sarcolemma barriers together with a MTT that is too short for complete Hb‐O_2_ off‐loading. This is supported by the need for an infinitesimal PO_2_ gradient for O_2_ to diffuse from the sarcoplasm to cytochrome c oxidase^66^ and the estimate that a mitochondrial PO_2_ of ~1 mmHg may be sufficient to support maximal mitochondrial respiration.^67^, ^68^ The remaining O_2_ may also represent muscle metabolism‐perfusion mismatch^69^, ^70^ and an inevitable lower O_2_ extraction from the blood perfusing the skin, connective tissue, fat and bone marrow of the leg causing venous admixture. In this context, the end‐capillary PO_2_, assessed using video microscopy, was found to be lower than the PO_2_ both in the venule (O_2_ microelectrode) and vein (blood gas) draining the muscle region of interest.^71^ Hence, the lowest femoral venous PO_2_ values of ~10 mmHg indicates an even lower end‐capillary PO_2_ in the capillaries adjacent to the most metabolically active muscle regions during maximal exercise, possibly approaching ~5 mmHg. Therefore, no matter which kind of limitation prevails, it is highly unlikely that leg O_2_ extraction fraction can improve much further, and that a theoretical threshold of ~95% exists because of the above diffusional and distributional limitations and barriers.

## 4 THE MECHANISMS EXPLAINING THE IMPROVEMENTS OF O_2_ EXTRACTION WITH TRAINING

The systemic ${\bar{O}}_{2}$ extraction fraction may increase through two main mechanisms with training: (a) by directing a higher fraction of ${\dot{Q}}_{\max}$ to the exercising muscles and (b) by increasing the peripheral O_2_ extraction fraction.

**Figure 7:**
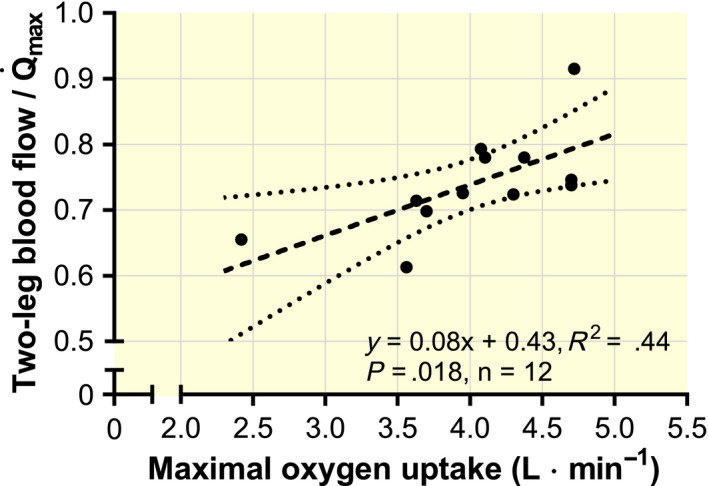
The fraction of maximal cardiac output ( ${\dot{Q}}_{\max}$) that is directed to the legs during maximal exercise (cycling) as a function of ${\dot{V}O}_{\text{2max}}$. The included studies measured ${\dot{Q}}_{\max}$ by using the indicator‐dilution method, Fick method or transpulmonary thermodilution, and leg blood flow was measured by thermodilution.^4^, ^5^, ^9^, ^11^, ^31^, ^51^, ^52^, ^53^, ^54^, ^55^, ^56^, ^57^ Note that the uppermost data point (0.915; ie, only 2.2 L**·**min^−1^ in calculated non‐leg blood flow) is supra‐physiological, but the correlation was similar after its exclusion (*R* ^2^ = .42)

Both in trained and untrained subjects, during exercise with a large muscle mass (such as running and cycling), the muscle‐specific blood flow (per unit of mass) is restrained as a result of sympathetically mediated vasoconstriction of peripheral vascular beds, caused by a limited ${\dot{Q}}_{\max}$.^9^, ^32^, ^48^ Even in “untrained” leg skeletal muscle, the reserve in vasodilatory capacity is very high and supports 2‐3 times larger blood flow per unit of mass, as observed during dynamic one‐legged knee extension.^72^ Simply increasing ${\dot{Q}}_{\max}$ (for instance, by training), without any peripheral adaptations, may increase the systemic ${\bar{O}}_{2}$ extraction fraction by two mechanisms. First, the recruitment of a larger portion of the already existing capillary network may reduce diffusion distances and thereby increase the O_2_ extraction. This additional recruitment may also serve to maintain MTT despite increased LBF. Second, a larger fraction of ${\dot{Q}}_{\max}$ will flow through the exercising muscles (Figure 7) because the non‐exercising tissue blood flow is independent of ${\dot{Q}}_{\max}$ in healthy young subjects (at ~6.4 L**·**min^−1^, see section 3.4).^4^, ^5^, ^9^, ^11^, ^31^, ^51^, ^52^, ^53^, ^54^, ^55^, ^56^, ^57^ Consequently, even without any peripheral adaptations, the systemic ${\bar{O}}_{2}$ extraction fraction may increase when ${\dot{Q}}_{\max}$ and LBF are elevated with training.

The peripheral O_2_ extraction depends on the interplay between several factors: (a) the kinetics of O_2_ off‐loading from Hb; (b) the erythrocyte MTT, which is determined by the blood flow, the capillary density, the capillary recruitment and the degree of matching of blood flow distribution to the metabolic demand; (c) the diffusional O_2_ conductance over the combined capillary wall, interstitium and sarcolemma barriers; and (d) the muscle oxidative capacity, the mitochondrial p50 and the mitochondrial activation.^10^, ^29^, ^73^

A right‐shifted O_2_‐Hb dissociation curve (elevated P_50_O_2_) increases the O_2_ extraction fraction in pump‐perfused dog muscle.^74^ A close relationship has also been demonstrated between O_2_ extraction fraction and in vivo P_50_O_2_ in humans during exercise.^29^ Very few of the studies included in the present analysis reported the in vivo P_50_O_2_, but it was possible to calculate it from the other blood gas parameters using Kelman’s Equation ^75^ after assuming a femoral venous blood temperature of 39.0°C at maximal exercise.^9^, ^55^, ^76^ Based on 15 of the studies presented in Table 2, the P_50_O_2_ was linearly associated with leg O_2_ extraction fraction (*R* ^2^ = .27; *n* = 15; *P* = .048). Despite this relationship, a high P_50_O_2_ does not seem to be compulsory to achieve high O_2_ extraction during whole‐body maximal exercise, as demonstrated in experiments using a small dose of carbon monoxide (carboxyhaemoglobin at 6%‐7%), which left‐shifts the ODC without a negative impact on O_2_ extraction fraction.^56^

Increased MTT has the potential to increase O_2_ extraction, but whether this occurs after endurance training is determined by the balance between the changes in blood flow and the capillary blood volume. Capillary density typically improves by 10‐30% after 4‐24 weeks of endurance training,^77^, ^78^, ^79^ which is similar to the changes in ${\dot{V}O}_{\text{2max}}$ for this training duration.^78^, ^79^, ^80^ Moreover, cross‐sectional data indicate a similar difference in capillary density to that of ${\dot{V}O}_{\text{2max}}$ between untrained and endurance trained men.^41^ Therefore, the capillary growth probably maintains the MTT despite elevated ${\dot{Q}}_{\max}$ and peripheral blood flow after training. In support, similar improvements in arm blood flow and capillary density have been observed after a period of arm training, causing no change in the calculated MTT.^47^ The arm O_2_ extraction fraction was increased in the same study, suggesting that elevated MTT is not the primary mechanism by which O_2_ extraction is improved after training. However, this may differ between arms and legs (ie, small vs large muscle mass exercise). Moreover, in the calculation of MTT in the study mentioned above, full capillary recruitment was assumed. Therefore, even though the changes in capillary density and muscle blood flow share magnitudes after endurance training, the MTT may still be increased if the capillary recruitment is altered.

An increased capillary‐to‐fibre ratio after endurance training increases the number of contact points between the capillary and the muscle fibre. This increases the diffusional surface area that, according to the Fick law of diffusion, increases the diffusive flux in a directly proportional manner. Therefore, the capillary‐to‐fibre ratio is regarded as a critical determinant of O_2_ diffusion from the erythrocytes to the cytoplasm.^81^, ^82^ As an example, a larger diffusional area and shorter diffusional distance are proposed to contribute to the higher O_2_ extraction fraction in the legs than in the arms during exercise.^29^ Moreover, if the capillary recruitment is changed with training, this may also affect the effective diffusional surface area similarly to de novo capillarization.

During whole‐body maximal exercise, the oxidative capacity of skeletal muscle exceeds the O_2_ delivery, as illustrated by the twofold higher ${\dot{V}O}_{2}$ per unit of muscle mass during dynamic one‐legged knee extension compared to cycling exercise (approximately 2.5 vs 20 kg active muscle mass, respectively).^10^, ^72^ Therefore, the leg muscles possess an oxidative reserve capacity at ${\dot{V}O}_{\text{2max}}$ during whole‐body exercise, which has frequently been used as an argument to indicate that the large improvements in mitochondrial and capillary networks after endurance training are likely only crucial for improvements in endurance performance and do not affect the limiting factors to ${\dot{V}O}_{\text{2max}}$.^83^ In support of this view, the calculated ${\bar{O}}_{2}$ extraction fraction is maintained or increases after prolonged bed rest (3‐6 weeks), although a substantial reduction in mitochondrial volume density occurs.^84^, ^85^ However, the ${\bar{O}}_{2}$ extraction fraction depends on the interactions between several factors. For instance, by acutely decreasing ${\dot{Q}}_{\max}$ and LBF using β‐adrenergic blockade, $a\text{-}\bar{v}O_{2}$ difference and a‐v_f_O_2_ difference increase during submaximal and maximal exercise, facilitated by increased erythrocyte MTT.^86^, ^87^ This is substantiated by the positive relationship between the ratio of OXPHOS/O_2_ delivery and the leg O_2_ extraction fraction,^10^ meaning that the balance between muscle oxidative capacity and blood flow (ie, oxidative capacity and MTT) is more critical for O_2_ extraction than any of these factors alone. Therefore, as bed rest reduces ${\dot{Q}}_{\max}$ dramatically but causes only a minor change in capillary density,^84^, ^85^ the MTT is elevated, and the ratio of OXPHOS/ O_2_ delivery is probably the same, in favour of increased or maintained ${\bar{O}}_{2}$ extraction fraction. In contrast, by changing the exercise mode from upright to supine cycling after bed rest, which preserves ${\dot{Q}}_{\max}$ at the pre‐bed rest level, the calculated $a\text{-}\bar{v}O_{2}$ difference is decreased (154 to 120 mL **·** L^–1^).^88^ Similarly, after a dog gastrocnemius muscle was immobilized for 3 weeks, followed by electrical stimulation to ${\dot{V}O}_{\text{2max}}$ while being pump perfused to receive a similar O_2_ delivery as a control muscle, the O_2_ extraction fraction was dramatically reduced.^82^ Therefore, muscle oxidative capacity seems to play a role in determining O_2_ extraction, and the bed rest studies need to be evaluated carefully because of the consequences for peripheral MTT.

If ${\bar{O}}_{2}$ extraction fraction improves after endurance training, is probably affected by the balance between central and peripheral adaptations. For instance, after 2 weeks of high‐intensity interval training that elevated the cytochrome c oxidase activity by 20% but caused no change in ${\dot{Q}}_{\max}$, ${\dot{V}O}_{\text{2max}}$ was increased by 8% and was entirely attributed to the improved systemic (calculated $a\text{-}\bar{v}O_{2}$ difference) and leg (increased deoxyhaemoglobin and decreased tissue oxygenation index in Vastus Lateralis, assessed using NIRS) O_2_ extraction.^89^ However, after 3‐8 weeks of endurance training, improvements in ${\dot{Q}}_{\max}$ explain almost the entire increase in ${\dot{V}O}_{\text{2max}}$, as indicated by meta‐regression.^44^ If the training lasts longer (>8 weeks), enhancements of ${\dot{Q}}_{\max}$ decelerate and improvements in $a\text{-}\bar{v}O_{2}$ difference are again evident.^44^, ^90^ Therefore, the peripheral adaptations are probably just sufficient to counteract the “negative influence” of elevated ${\dot{Q}}_{\max}$ and LBF on MTT in periods with large central adaptations, and improvements in ${\bar{O}}_{2}$ extraction fraction is likely only evident when the peripheral adaptations largely surpass those of the central circulation. This can be substantiated by findings from one‐legged endurance training that induces robust peripheral adaptations without stimulating the central circulation substantially and commonly improves leg a‐v_f_O_2_ difference by 5‐10 mL **·** L^–1^.^30^, ^45^

The mitochondrial volume density can differ by as much as 150% between untrained and well‐trained individuals in extreme cases (eg, ~4 vs ~10 vol. %)^91^, ^92^ and can improve by as much as ~40%‐55% after 6 weeks of endurance training in previously sedentary individuals.^38^, ^93^, ^94^ Why does this disproportionate adaptation occur when the muscle already possesses an oxidative reserve capacity? Does it have any physiological meaning for ${\dot{V}O}_{\text{2max}}$ or is it only important for improvements in, for example, fat oxidation^95^ and the lactate threshold,^96^ thus improving endurance?

Although an impressive increase in leg O_2_ extraction fraction from 72% to 82% has been reported after only 9 weeks of intense endurance training in previously sedentary subjects,^12^ we propose that remarkable increases in muscle oxidative capacity are needed to achieve the outstanding leg O_2_ extraction fraction observed in elite athletes (close to 95%).^29^, ^97^ By analogy, the oxidative reserve capacity may act as a “bottomless pit”, keeping the myoglobin SO_2_ and intracellular PO_2_ low. This, in turn, maintains the PO_2_ gradient between the capillary and the muscle cell, promoting O_2_ diffusion and O_2_ extraction even at a very low capillary PO_2_.

Emerging evidence suggests that the mitochondrial volume density is increased while their intrinsic OXPHOS (OXPHOS divided by mitochondrial volume density or citrate synthase activity) is unchanged^89^, ^98^, ^99^ and sometimes even reduced^94^, ^100^ after training. Since the mitochondrial respiratory rate and the ex vivo mitochondrial p50 increase in parallel,^10^, ^73^ the unchanged or reduced intrinsic OXPHOS after training may permit an increased OXPHOS per unit of muscle mass while preserving (or increasing) the mitochondrial O_2_ affinity (ie, by keeping the mitochondrial p50 low).^73^ Thus, a large pool of mitochondria with high O_2_ affinity may preserve mitochondrial activation at low O_2_ availability (low capillary PO_2_) and promote peripheral O_2_ extraction, but is yet to be experimentally tested. Moreover, the subsarcolemmal mitochondrial population increases relatively more than the intermyofibrillar population after endurance training.^93^, ^94^ These mitochondrial clusters in close proximity to the capillaries may, speculatively, amplify the O_2_ concentration gradient, shorten the diffusional distance and, thus, promote O_2_ diffusion across the sarcolemma^101^ and enable further O_2_ extraction at the end of the capillaries.

As shown in Figure 6C, a subject’s ${\dot{V}O}_{\text{2max}}$ becomes gradually less sensitive to adaptations improving diffusion when ${\dot{V}O}_{\text{2max}}$ is already high. Therefore, to raise the O_2_ extraction fraction even slightly (eg, 2%), it is likely that more substantial improvement in peripheral adaptations is needed. However, a change in leg O_2_ extraction fraction from, for example, 93% to 95% would only have a small impact on whole‐body ${\dot{V}O}_{\text{2max}}$: for an athlete with a ${\dot{V}O}_{\text{2max}}$ of 5 L**·**min^−1^, a two‐LBF of 24 L**·**min^−1^ ( ${\dot{Q}}_{\max}$: ~31 L**·**min^−1^) and an CaO_2_ of 190 mL **·** L^–1^, the ${\dot{V}O}_{\text{2max}}$ would only increase by ~90 mL **·** min^–1^ (1.8%). In comparison, an increase of 1 L**·**min^−1^ in two‐LBF would increase ${\dot{V}O}_{\text{2max}}$ by ~170 mL **·** min^–1^ (3.4%) if all other factors remained the same.

## 5 STUDY CONSIDERATIONS

The data were collected from several research groups and published over six decades (1958‐2017) using a variety of gas analysers, flow sensors, methods to determine blood O_2_ content and PO_2_, and several procedures to analyse the indicator‐dilution and blood temperature curves for ${\dot{Q}}_{\max}$ and LBF measurements respectively. Therefore, for a given ${\dot{V}O}_{\text{2max}}$, the between‐subject variability presented here may be overestimated. Moreover, several different averaging strategies for ${\dot{V}O}_{2}$ and the associated variables have likely been applied (rarely stated in the manuscripts). Despite these potential sources of noise, in general, the studies’ mean values converged to similar values. The fact that, despite the combination of several measurements with distinct methods (such as pulmonary gas exchange, thermodilution and blood gas analyses), the integrations of the obtained values fitted into the physiological range and agreed between studies, demonstrates the quality of these studies and the robustness of the analysis presented here.

## 6 CONCLUSION AND PERSPECTIVE

**Figure 8:**
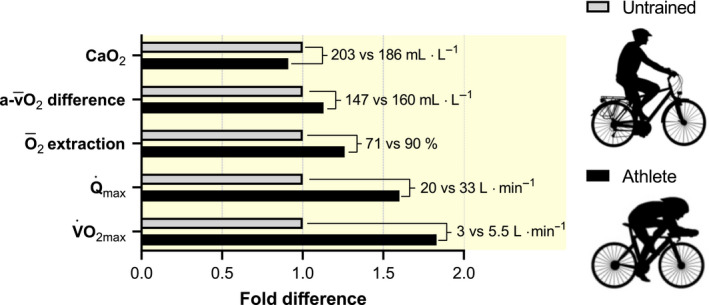
A comparison of an untrained individual and an elite endurance athlete with maximal oxygen uptakes ( ${\dot{V}O}_{\text{2max}}$) of 3.0 and 5.5 L**·**min^−1^ respectively. The maximal cardiac output ( ${\dot{Q}}_{\max}$), systemic O_2_ extraction fraction ( ${\bar{O}}_{2}$ extraction), arterial to mixed venous O_2_ difference ( $a\text{-}\bar{v}O_{2}$ difference) and arterial O_2_ content (CaO_2_) were calculated using the regression equations presented in Figure 1

In conclusion, measurements of ${\dot{Q}}_{\max}$ and LBF show that O_2_ delivery is the primary determinant of whole‐body and limb ${\dot{V}O}_{\text{2max}}$. However, we also show that a very high O_2_ extraction fraction contributes to the remarkably high ${\dot{V}O}_{\text{2max}}$ in well‐trained individuals and elite endurance athletes. To reinforce this conclusion we can, using the regression lines established in the present investigation, compare a typically sedentary subject and an elite endurance athlete with a large difference in ${\dot{V}O}_{\text{2max}}$ (3.0 vs 5.5 L**·**min^−1^): the elite athlete has a 1.83‐fold higher ${\dot{V}O}_{\text{2max}}$, a 1.60‐fold higher ${\dot{Q}}_{\max}$ and a 1.26‐fold higher ${\bar{O}}_{2}$ extraction fraction (Figure 8). However, because of the lower CaO_2_, the $a\text{-}\bar{v}O_{2}$ difference is only 1.13‐fold higher in the elite athlete. This also stresses that $a\text{-}\bar{v}O_{2}$ difference and ${\bar{O}}_{2}$ extraction fraction cannot be used interchangeably when evaluating central versus peripheral limitations to ${\dot{V}O}_{\text{2max}}$. Finally, the limitations for whole‐body ${\dot{V}O}_{\text{2max}}$ change with training status, with an accentuated O_2_ delivery limitation and conversely a decreasing O_2_ diffusional limitation with increasing ${\dot{V}O}_{\text{2max}}$.

## CONFLICT OF INTEREST

The authors declare no conflict of interest, financial or otherwise.

## AUTHOR CONTRIBUTIONS

Conception and design of the investigation: ØS, JH, CC, JALC. Literature search and analysis of data: ØS. Interpretation of data: ØS, JH, CC, JALC, BR. Writing the first draft of the manuscript: ØS. Revising and approving the final version: ØS, JH, CC, JALC, BR.

## Supporting information

Supplementary Material

## Data Availability

Data sharing is not applicable to this article, as no new data were created in this study.
