# Supplementary material for: Contribution of oxygen extraction fraction to maximal oxygen uptake in healthy young men
Source: Acta Physiol (Oxf). 2020 May 30;230(2):e13486. doi: 10.1111/apha.13486 (PMC7540168; doi:10.1111/apha.13486)

**Supporting information**

**Contribution of oxygen extraction fraction to maximal oxygen uptake in healthy young men**

**Supporting information Fig. 1:** The relationships between pulmonary maximal oxygen uptake standardised to body weight and systemic oxygen extraction fraction (a), arterial to mixed venous oxygen difference (a-$\bar{v}$O_2_ difference; b), leg oxygen extraction fraction (c), and arterial to femoral venous oxygen difference (a-v_f_O_2_ difference; d). Data are mean values (± 95% confidence limits, where available) from studies reported in Table 1 (systemic responses) and Table 2 (peripheral responses) of the manuscript.


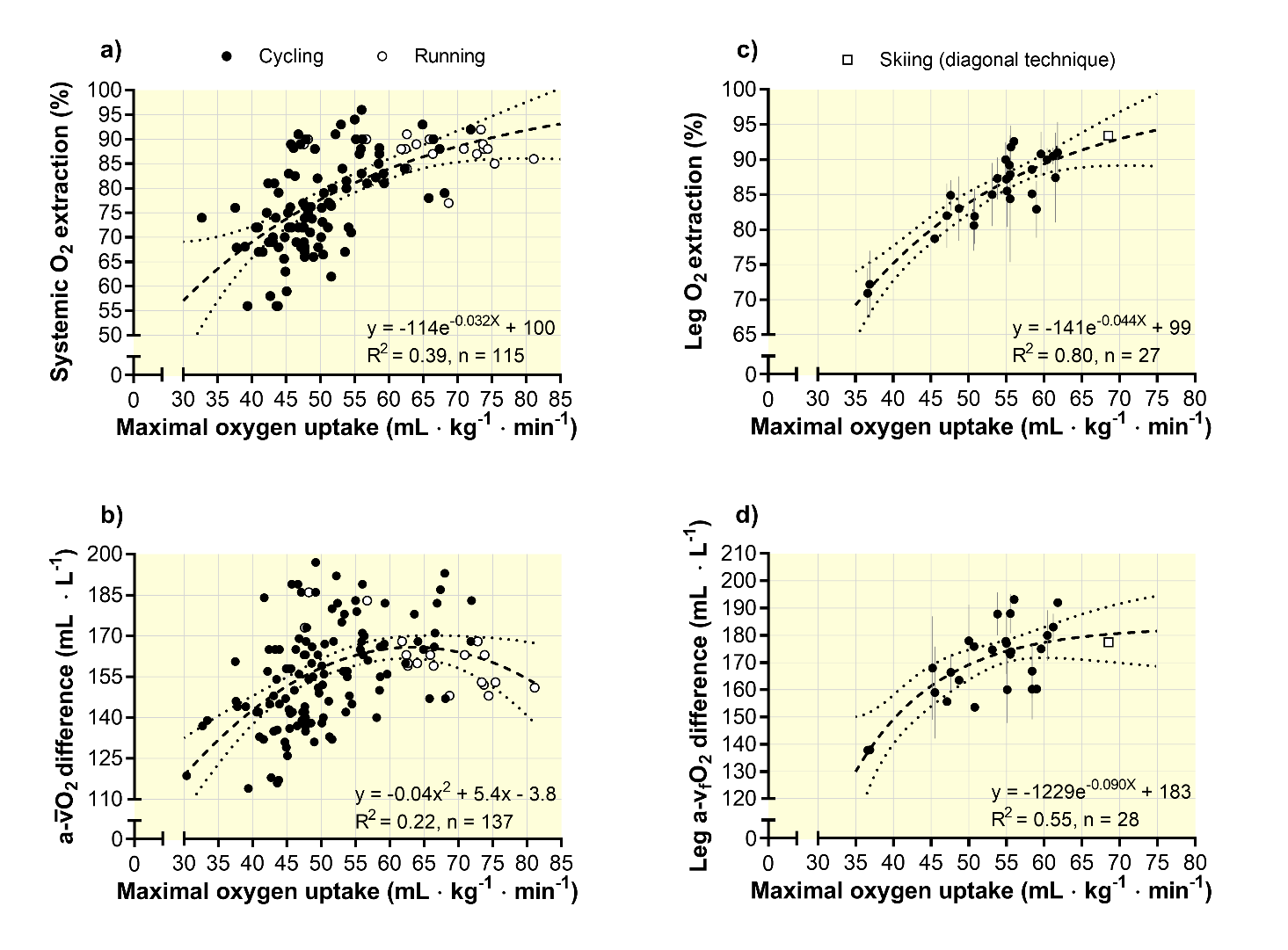


**Supporting information Fig. 2:** The relationship between pulmonary maximal oxygen uptake standardised to body weight and the equilibration index Y. Black and white symbols denote cycling and diagonal skiing, respectively. Data are from studies reported in Table 2 of the manuscript.


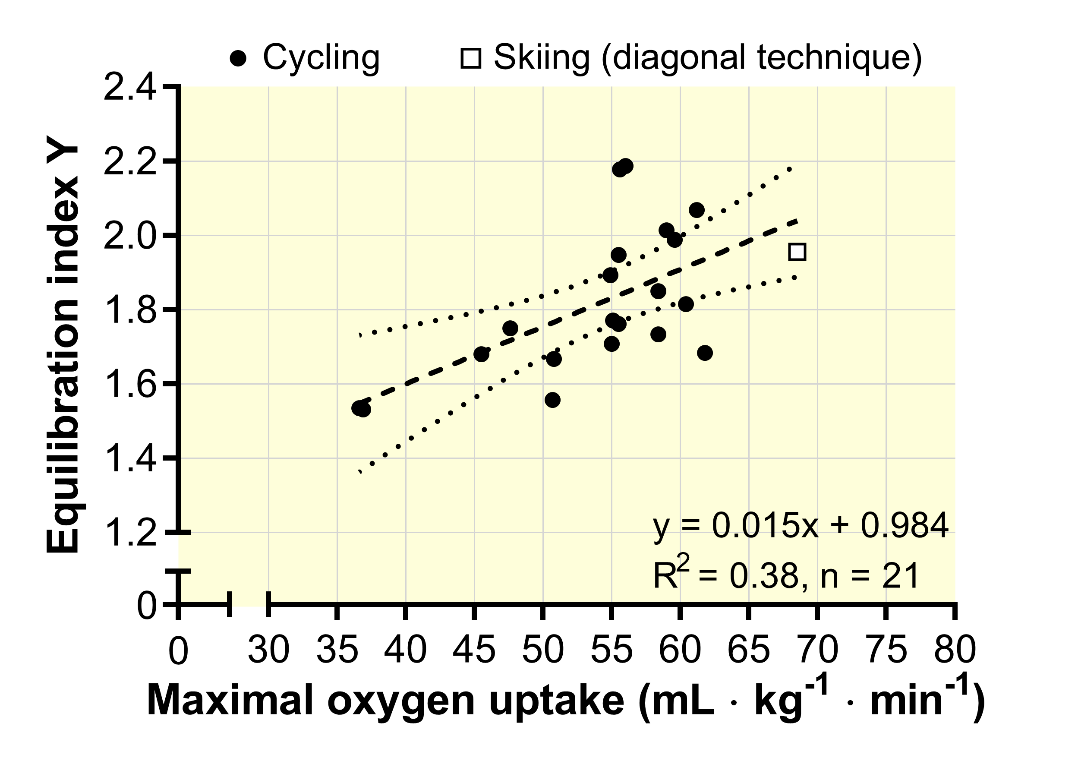

Supplement: Supplementary file 1 — Supplementary Material [file APHA-230-e13486-s001.docx]
